# Persistent effects of Libby amphibole and amosite asbestos following subchronic inhalation in rats

**DOI:** 10.1186/s12989-016-0130-z

**Published:** 2016-04-15

**Authors:** Stephen H. Gavett, Carl U. Parkinson, Gabrielle A. Willson, Charles E. Wood, Annie M. Jarabek, Kay C. Roberts, Urmila P. Kodavanti, Darol E. Dodd

**Affiliations:** 1 National Health and Environmental Effects Research Laboratory, U.S. Environmental Protection Agency, Research Triangle Park, NC 27711 USA; 2 The Hamner Institutes for Health Sciences, Research Triangle Park, NC 27711 USA; 3 Experimental Pathology Laboratories, Inc. (EPL®), Research Triangle Park, NC 27711 USA; 4 National Center for Environmental Assessment, U.S. Environmental Protection Agency, Research Triangle Park, NC 27711 USA

**Keywords:** Asbestos, Libby amphibole, Amosite, Inhalation, Inflammation, Fibrosis, Adenoma, Carcinoma, Dosimetry, Risk assessment

## Abstract

**Background:**

Human exposure to Libby amphibole (LA) asbestos increases risk of lung cancer, mesothelioma, and non-malignant respiratory disease. This study evaluated potency and time-course effects of LA and positive control amosite (AM) asbestos fibers in male F344 rats following nose-only inhalation exposure.

**Methods:**

Rats were exposed to air, LA (0.5, 3.5, or 25.0 mg/m^3^ targets), or AM (3.5 mg/m^3^ target) for 10 days and assessed for markers of lung inflammation, injury, and cell proliferation. Short-term results guided concentration levels for a stop-exposure study in which rats were exposed to air, LA (1.0, 3.3, or 10.0 mg/m^3^), or AM (3.3 mg/m^3^) 6 h/day, 5 days/week for 13 weeks, and assessed 1 day, 1, 3, and 18 months post-exposure. Fibers were relatively short; for 10 mg/m^3^ LA, mean length of all structures was 3.7 μm and 1 % were longer than 20 μm.

**Results:**

Ten days exposure to 25.0 mg/m^3^ LA resulted in significantly increased lung inflammation, fibrosis, bronchiolar epithelial cell proliferation and hyperplasia, and inflammatory cytokine gene expression compared to air. Exposure to 3.5 mg/m^3^ LA resulted in modestly higher markers of acute lung injury and inflammation compared to AM. Following 13 weeks exposure, lung fiber burdens correlated with exposure mass concentrations, declining gradually over 18 months. LA (3.3 and 10.0 mg/m^3^) and AM produced significantly higher bronchoalveolar lavage markers of inflammation and lung tissue cytokines, Akt, and MAPK/ERK pathway components compared to air control from 1 day to 3 months post-exposure. Histopathology showed alveolar inflammation and interstitial fibrosis in all fiber-exposed groups up to 18 months post-exposure. Positive dose trends for incidence of alveolar epithelial hyperplasia and bronchiolar/alveolar adenoma or carcinoma were observed among LA groups.

**Conclusions:**

Inhalation of relatively short LA fibers produced inflammatory, fibrogenic, and tumorigenic effects in rats which replicate essential attributes of asbestos-related disease in exposed humans. Fiber burden, inflammation, and activation of growth factor pathways may persist and contribute to lung tumorigenesis long after initial LA exposure. Fiber burden data are being used to develop a dosimetry model for LA fibers, which may provide insights on mode of action for hazard assessment.

**Electronic supplementary material:**

The online version of this article (doi:10.1186/s12989-016-0130-z) contains supplementary material, which is available to authorized users.

## Background

The vermiculite mine near Libby, in northwestern Montana was the world’s leading source of vermiculite for 70 years until its closure in 1990 [1]. Vermiculite is used for insulation, as an absorbent material, and as a soil conditioner, with applications in the construction, agricultural, horticultural, and industrial markets. The Libby vermiculite ore coexists with a complex array of amphibole mineral types referred to as Libby amphibole (LA) asbestos, primarily winchite, richterite, and tremolite, which have a range of crystal morphologies ranging from asbestiform to acicular or prismatic [2]. Occupational exposure to LA is associated with significantly increased risk of respiratory disease, including non-malignant respiratory disease (such as localized and diffuse pleural thickening, asbestosis, and autoimmune disease), lung cancer, and mesothelioma, compared to background rates within the U.S. population [3–11]. Risks of non-occupational exposure to LA have also been reported. For example, pleural abnormalities (calcifications, thickenings, or plaques) were identified on chest radiographs in 18 % of 6668 persons who lived or worked in the Libby area for at least 6 months before 1991, with greater prevalence among those with more types or routes of exposure [12, 13]. Outside of Libby, exposure to LA is a continuing risk since the contaminated vermiculite ore was shipped to locations around the nation for processing and used in a variety of applications, especially attic insulation which may be present in homes throughout the United States [1].

Several previous studies have investigated the fibrotic and tumorigenic effects of amphibole asbestos in rodents [14, 15], although no inhalation studies have specifically assessed the potency of LA asbestos present at the Libby vermiculite mine. The goals of this study were to examine internal dose, time course of pathological effects, and potential mode of action (MOA) of inhaled LA in rats, and to improve the scientific basis for the risk assessment of asbestos-contaminated communities. Inhalation exposure scenarios were adapted from testing strategies recommended for toxicity assessment of fibrous particles [16]. Effects of LA and amosite (AM), a known fibrogenic and carcinogenic amphibole asbestos fiber [14], were initially evaluated in rats exposed for 10 days in a nose-only inhalation study. This short-term study provided data on early fiber effects and guided exposure concentrations for a subsequent long-term stop-exposure study in which rats were exposed by inhalation to LA or AM for 13 weeks and then evaluated at different time points up to 18 months post-exposure. The biological potency of inhaled LA and AM over the near-life span of the rat was compared in terms of lung inflammation and respiratory tract pathology, including tumor formation. Since inflammation is thought to be a key event leading to respiratory tract fibrosis and tumorigenesis [17, 18], several markers and pathways of inflammation were examined in lung tissue, including mRNA expression of inflammasome pathway components and pro-inflammatory cytokines, protein levels of pro-inflammatory or pro-allergic cytokines, and protein levels of Akt and mitogen-activated protein kinases/extracellular signal-regulated kinases (MAPK/ERK) pathway components. Additional fiber burden data from the satellite component for time-course data in this study are being used to develop a dosimetry model of amphibole fiber deposition, clearance, and retention in the respiratory tract, pleura, and lymph nodes. The dosimetry model will enable calculation of different dose metrics and translation of dose across different exposure scenarios for linkage of laboratory animal data to epidemiological data.

## Results

### Short-term inhalation study

**Fig. 1:**
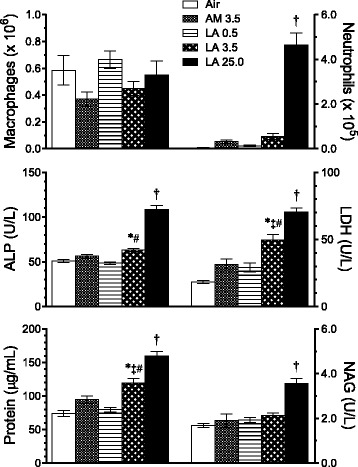
Short-term inhalation study: lung inflammation markers in bronchoalveolar lavage fluid (BALF) immediately after final exposure to AM or LA for 10 days (5 days/week for 2 weeks; 6 h/day). Numbers in legend represent target mass concentrations (mg/m^3^). Values represent mean ± SE of BALF macrophages and neutrophils (top panels) and concentrations of ALP, LDH, protein, and NAG in BALF supernatant (middle and lower panels) (*n* = 7 rats/group). *P* < 0.05 † vs. all other groups; * vs. air control; # vs. LA 0.5; ‡ vs. AM 3.5

Airway inflammation was assessed immediately after the final day of nose-only inhalation exposure for 10 days (5 days/week for 2 weeks; 6 h/day) to air alone or target concentrations of 3.5 mg/m^3^ AM or 0.5, 3.5, or 25.0 mg/m^3^ LA. Numbers of bronchoalveolar lavage fluid (BALF) alveolar macrophages were not significantly affected by exposure, whereas neutrophils were observed in all AM and LA exposure groups, and were significantly higher in the 25.0 mg/m^3^ LA-exposed group compared to all other groups (Fig. 1). As a percentage of total BALF cells, neutrophils were significantly higher in the 3.5 (10 %) and 25.0 (47 %) mg/m^3^ LA groups but not the 3.5 mg/m^3^ AM group (7 %) compared to air controls. Lymphocytes were less than 1 % of total BALF cells in all groups (not shown). Compared with all other groups, the 25.0 mg/m^3^ LA group also had significantly greater levels of BALF supernatant protein (marker of lung edema and epithelial cell injury), lactate dehydrogenase (LDH; marker of cytotoxicity), N-acetyl β-D glucosaminidase (NAG; marker of macrophage lysosomal activation), and alkaline phosphatase (ALP; marker of fibrosis [19]) (Fig. 1). Protein and LDH levels were also significantly higher in the 3.5 mg/m^3^ LA-exposed group compared with air-, 0.5 mg/m^3^ LA-, and 3.5 mg/m^3^ AM-exposed groups, indicating greater lung toxicity of LA compared with AM at the same mass concentration.

**Fig. 2:**
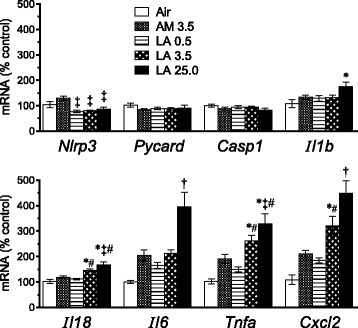
Short-term inhalation study: transcriptional markers of apoptosis and inflammation in lung samples after final exposure to AM or LA for 10 days. Results show relative mean values ± SE of lung tissue mRNA for inflammasome pathway components (*Nlrp3*, *Pycard*, *Casp1*), downstream cytokines (*Il1b* and *Il18*), and other pro-inflammatory cytokines (*Il6*, *Tnfa*, *Cxcl2*) as determined by RT-qPCR (*n* = 7 rats per group). *P* < 0.05 † vs. all other groups; * vs. air control; # vs. LA 0.5; ‡ vs. AM 3.5

Components of the NLRP3 inflammasome pathway, including NALP3 (NACHT, LRR and PYD domains-containing protein 3), encoded by the *Nlrp3* (NOD-like receptor family, pyrin domain containing 3) gene, PYCARD (pyrin domain caspase recruitment domain (CARD); also known as apoptosis-associated speck-like protein containing a CARD (ASC)), and caspase-1 (Casp1), were initially recognized to play a role in inflammation and apoptosis in response to microbial infection [20], and may contribute to the development of fibrosis after asbestos exposure [21, 22]. Caspases in turn can activate pro-inflammatory cytokines including interleukin-1β (IL-1β) and IL-18 [20]. However, immediately after 10 days exposure to AM or LA, lung tissue mRNA expression of *Nlrp3*, *Pycard*, and *Casp1* were unchanged in comparison to air-exposed controls (Fig. 2). Expression of *Il1b* and *Il18* were only modestly higher in the 25.0 mg/m^3^ LA group. In contrast, expression of several other pro-inflammatory cytokines, including *Il6*, *Tnfa* (tumor necrosis factor-α; TNF-α), and *Cxcl2* (chemokine (C-X-C motif) ligand 2, also known as macrophage inflammatory protein 2) were 2- to 4-fold higher in groups exposed to LA at 3.5 or 25.0 mg/m^3^, but were not significantly higher in the AM group compared to controls (Fig. 2). Expression of *Ifng* (interferon-γ; IFN-γ), which has been shown to inhibit activation of the NALP3 inflammasome [23], was unchanged in all groups (not shown).

**Fig. 3:**
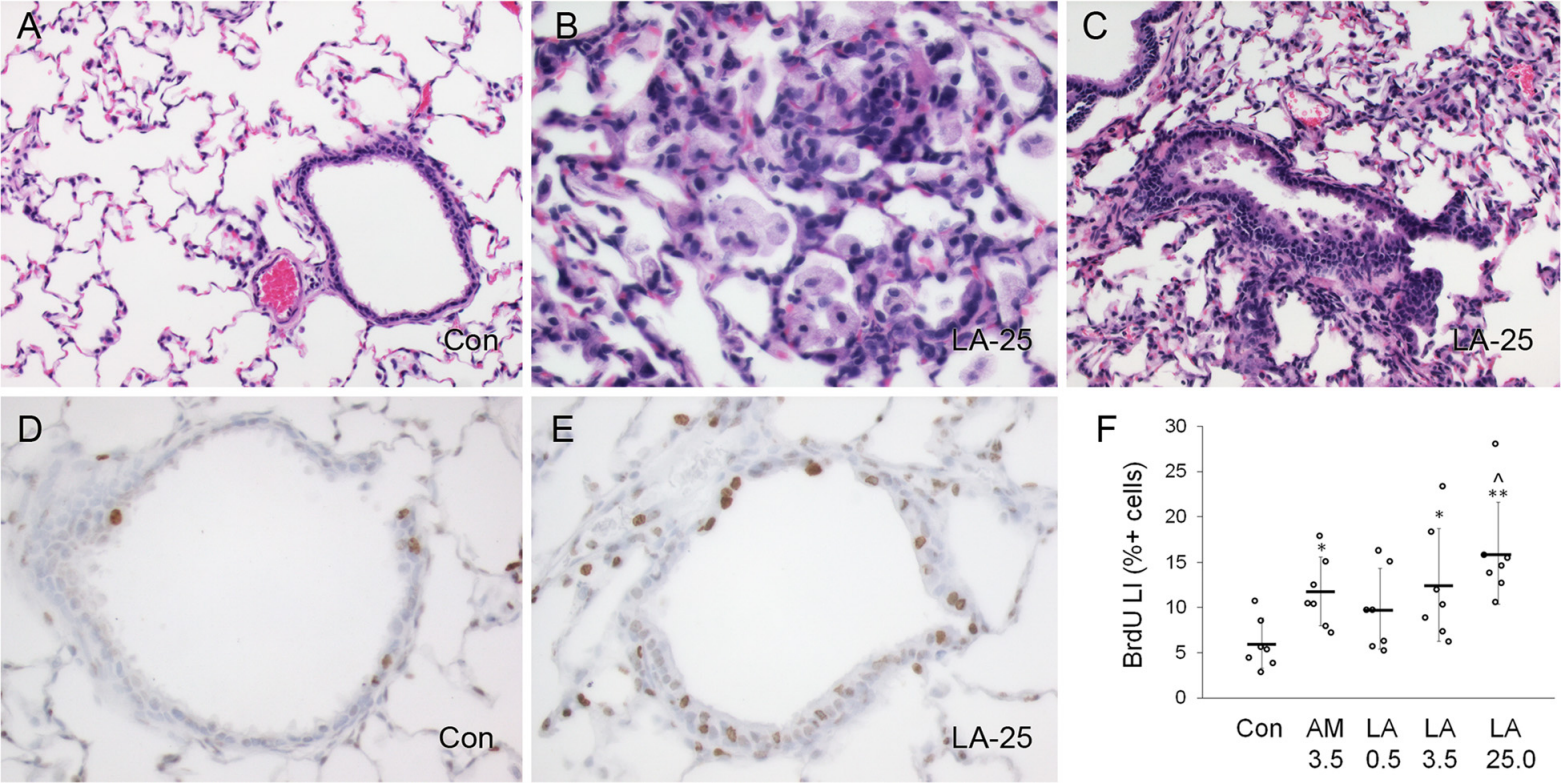
Short-term inhalation study: histopathologic effects and terminal bronchiolar epithelial cell proliferation 4 days after final exposure to AM or LA for 10 days. **a**–**c**, Representative images of normal terminal bronchiole and surrounding alveoli from air control (Con) group (**a**), mixed cell alveolar inflammation, predominantly macrophages and neutrophils (**b**), and bronchiolar epithelial hyperplasia (**c**). Slides were stained with hematoxylin and eosin. **d**–**f**, Immunostaining of cells with BrdU. Representative images of terminal bronchioles from Con (**d**) and high concentration LA (25 mg/m^3^ target) (**e**) groups. Positively labeled cells are indicated by brown nuclei; background staining used hematoxylin. **f**, Percentage of positive BrdU-labeled cells (labeling index, LI), shown as individual LI values (*circles*), mean values (*horizontal bars*), and standard error (*vertical bars*) for each group. *P < 0.05 and **P < 0.01 compared to air control (Con) group; ^ *P* < 0.01 by linear trend test among LA dose groups. Images were taken at 20x (**a**, **c**) or 40x (**b**, **d**, **e**) objective magnification

Lung histopathology was assessed 4 days after the 10-day exposure (Table 1). Alveolar inflammation was observed in all AM- and LA-exposed groups. The severity of this change was greater in the 3.5 and 25.0 mg/m^3^ LA groups compared to the AM group, similar to BALF injury markers (LDH and protein) (Fig. 1). Alveolar inflammation was characterized by infiltration of macrophages and lesser numbers of neutrophils and lymphocytes around alveoli, alveolar ducts, and terminal bronchioles (TBs) (predominant centriacinar distribution) (compare Fig. 3a and b). Inflammation was often associated with the presence of rod-shaped foreign bodies (consistent with asbestos fibers) in the cytoplasm of alveolar macrophages. In more severely affected areas, cytotoxicity was evident and characterized by pyknotic nuclei and karyorrhectic debris.

**Table 1:** Lung histopathology (left lobe) after exposure to AM or LA for 10 days

	Air control	AM	LA	LA	LA
Target mass concentration (mg/m^3^)	0.00	3.50	0.50	3.50	25.00
Actual mass concentration (mg/m^3^)	0.08 ± 0.18	3.67 ± 1.58	0.53 ± 0.11	3.59 ± 0.91	26.76 ± 9.11
Foreign Body^a^	0	6*	2	6*	7*
Alveolus Inflammation^b^	0	7 (1.0)*	6 (0.9)*	7 (2.0)*	7 (2.0)*
Focal Chronic Inflammation^c^	3 (0.4)	3 (0.4)	5 (0.7)	1 (0.1)	0
Bronchiole Epithelial Hyperplasia	0	0	0	0	5 (0.7)*
Interstitial Fibrosis^d^	0	4 (0.6)	0	3 (0.4)	7 (1.0)*

Rats were exposed to AM or LA 6 h/day, 5 days/week for 2 weeks and evaluated 4 days after final exposure. Mass concentration data shown are mean values ± SD of daily mass concentrations. Other values represent the number of animals with a finding in each group (*n* = 7 per group). Average lesion severity scores for all rats in each group (shown in parentheses) were graded as follows: 1 = minimal, 2 = mild, 3 = moderate, 4 = marked, and 5 = severe. *P < 0.05 vs. air control by Fisher’s Exact Test. ^a^Consistent with fibers; not graded for severity. ^b^Predominantly macrophages with lesser numbers of neutrophils and lymphocytes, often associated with foreign bodies. ^c^Small aggregates of alveolar macrophages, often subpleural; distinct from the predominantly centriacinar populations of mixed inflammatory cells observed with “alveolus inflammation”. ^d^Confirmed on Masson’s trichrome stain

The incidences of bronchiolar epithelial hyperplasia and interstitial fibrosis were also higher in the 25.0 mg/m^3^ LA group compared to the control air group (Table 1). Bronchiolar hyperplasia was indicated by an increased number of epithelial cells in the TBs with variable degrees of karyomegaly and anisokaryosis (Fig. 3c). Interstitial fibrosis was characterized by fibroblasts and collagen deposition along alveolar walls (most prominent at branch points into alveolar sacs) and within areas of granulomatous inflammation. The increase in collagen was confirmed by positive staining with Masson’s trichrome (not shown). Occasional areas of fibrosis also contained foci of multilamellar mineralization.

Epithelial cell proliferation within TBs was assessed by 5-bromo-2′-deoxyuridine (BrdU) labeling (compare Fig. 3d and e). BrdU labeling index (LI) was increased in a concentration-dependent manner in LA-exposed rats (linear dose trend *P* = 0.001), and was significantly greater for the 3.5 mg/m^3^ AM and 3.5 and 25.0 mg/m^3^ LA groups compared to the air control group (adjusted *P* < 0.05 for all) (Fig. 3f). Comparable BrdU incorporation at the same mass concentration of AM and LA contrasted with greater lung inflammation for LA than AM (Fig. 1, Table 1). The latter findings suggest that AM and LA exposure resulted in a similar degree of terminal bronchiolar cytotoxicity, which may occur independently from airway inflammation, at least in short-term exposures [24].

Based on the overall inflammatory response in BALF, expression of pro-inflammatory cytokines, and histopathological effects in the lung, it was determined that the 13-week inhalation study should incorporate a 10-fold range of LA mass concentrations, from 1.0 to 10.0 mg/m^3^ LA, and that the AM concentration of 3.3 mg/m^3^ should match the intermediate LA concentration.

### 13-week inhalation exposure characterization

Daily mass concentrations of the air control, AM (3.3 mg/m^3^), and LA (1.0, 3.3, and 10.0 mg/m^3^) exposures closely matched target levels in the 13-week inhalation study (Table 2). Count median aerodynamic diameters and geometric standard deviation (σ_g_), as determined weekly by an aerosol particle sizer (APS), were similar for all asbestos exposures, averaging 0.9–1.1 μm and 1.4–1.5, respectively. The APS indicated a higher concentration of particles in the AM exposure compared with the same mass concentration of LA, but examination of all asbestos structures (any dimension ≥ 0.2 μm) with a scanning electron microscope (SEM) showed that the number concentrations for the AM and LA 3.3 mg/m^3^ exposures were comparable (3101/cm^3^ and 3728/cc, respectively). Examination of all structure size distributions by SEM showed slightly greater lengths (L) and aspect ratios (L/diameter (D)) for the LA 3.3 mg/m^3^ exposure (mean L: 3.3 μm; L/D: 8.7) compared with the AM exposure (mean L: 1.9 μm; L/D: 5.8). Lengths and L/D of LA structures tended to increase at higher mass concentrations (mean L: 3.7 μm; L/D: 9.5 at 10.0 mg/m^3^ LA). The greater L and L/D of LA compared to AM structures resulted in a greater proportion of structures classified as fibers as defined by the World Health Organization (WHO) (L ≥ 5 μm and L/D ≥ 3): 7.4 % of AM structures were WHO fibers compared with 18.6 % of LA structures at the same mass concentration (Table 2). Consequently, the AM exposure had only 44 % more WHO fibers/cc than the low LA exposure, despite having 3-fold greater mass and structure concentration. Within the WHO fiber class, L, D, and L/D were similar for all AM and LA exposures. There were few very long fibers (≥20 μm), which comprised 3–5 % of WHO fibers (approximately 1 % or less of all structures), and their concentration ranged up to 75 fibers/cc in the 10.0 mg/m^3^ LA exposure. These LA and AM fiber samples were overall relatively short in comparison to samples of AM or other asbestos types studied previously [15, 25].

**Table 2:** Characteristics of 13-week subchronic exposure

	Air control	AM	LA	LA	LA
Target mass concentration (mg/m^3^)	0.00	3.30	1.00	3.30	10.00
Actual mass concentration (mg/m^3^)	0.00 ± 0.16	3.32 ± 1.48	1.01 ± 0.45	3.34 ± 0.83	10.04 ± 2.04
APS: Count median aerodynamic diameter (μm)	1.41 ± 0.98	0.93 ± 0.04	1.05 ± 0.06	1.02 ± 0.03	1.13 ± 0.05
APS: σ_g_	2.01 ± 0.58	1.40 ± 0.08	1.51 ± 0.03	1.46 ± 0.02	1.52 ± 0.04
APS: Particles/cc	1 ± 3	404 ± 132	43 ± 23	171 ± 56	280 ± 96
SEM: All structures/cc	-	3101 ± 598	1158 ± 217	3728 ± 772	6718 ± 958
SEM: Total structures counted (*n*)	-	3218	2557	4517	2674
SEM: All structure lengths, L (μm)					
Mean	-	1.9 ± 0.1	2.6 ± 0.4	3.3 ± 0.4	3.7 ± 0.4
Median		1.0 ± 0.1	1.4 ± 0.4	2.2 ± 0.4	2.5 ± 0.4
Maximum		21.0 ± 7.1	22.0 ± 6.0	25.3 ± 5.0	27.5 ± 8.4
SEM: All structure diameters, D (μm)					
Mean	-	0.33 ± 0.01	0.38 ± 0.03	0.43 ± 0.02	0.44 ± 0.03
Median		0.29 ± 0.01	0.32 ± 0.02	0.36 ± 0.02	0.37 ± 0.03
Maximum		1.38 ± 0.85	2.14 ± 0.78	2.29 ± 0.52	2.56 ± 1.29
SEM: All structure aspect ratios (L/D)					
Mean	-	5.8 ± 0.5	7.0 ± 0.9	8.7 ± 0.9	9.5 ± 0.8
Median		3.1 ± 0.3	3.8 ± 0.7	5.6 ± 0.9	6.2 ± 0.7
Maximum		60.7 ± 16.4	69.5 ± 36.6	86.8 ± 41.2	85.5 ± 42.4
SEM: WHO fibers/cc (L/D ≥3; L ≥5 μm)	-	230 ± 56	159 ± 63	693 ± 199	1522 ± 304
SEM: Total WHO fibers counted (*n*)		237	349	838	601
Proportion of structures which are WHO fibers (%)	-	7.4	13.6	18.6	22.5
SEM: WHO fiber L (μm)					
Mean	-	8.9 ± 0.9	9.0 ± 0.8	9.0 ± 0.6	9.3 ± 0.7
Median		7.3 ± 0.9	7.6 ± 0.7	7.6 ± 0.4	7.7 ± 0.6
SEM: WHO fiber D (μm)					
Mean	-	0.41 ± 0.04	0.51 ± 0.09	0.49 ± 0.03	0.52 ± 0.05
Median		0.37 ± 0.04	0.44 ± 0.08	0.42 ± 0.03	0.45 ± 0.04
Maximum		0.80 ± 0.25	1.46 ± 0.72	1.66 ± 0.48	1.47 ± 0.39
SEM: WHO fiber aspect ratios (L/D)					
Mean	-	24.8 ± 3.7	22.1 ± 2.8	22.5 ± 2.4	22.3 ± 2.1
Median		21.5 ± 3.9	18.6 ± 2.6	18.7 ± 1.6	17.7 ± 1.9
SEM: Fibers, L ≥20 μm/cc (L/D ≥3)	-	7 ± 10	5 ± 6	26 ± 18	75 ± 52
Proportion of WHO fibers with L ≥20 μm (%)	-	3.0	3.2	3.8	4.8

Results shown are mean values ± SD of all daily mass concentrations (*n* = 66–67 per group) and values from weekly aerodynamic particle sizer (APS) and scanning electron microscopy (SEM) measurements (*n* = 12–14 per group)

### Tissue fiber burden

**Fig. 4:**
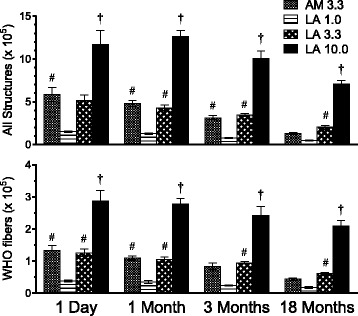
Long-term stop-exposure inhalation study: lung fiber burden. Numbers of all asbestos structures (any structure with L ≥0.2 μm; *top panel*) and WHO fibers (L ≥5 μm, L/D ≥3; *bottom panel*) recovered in five lung lobes (LRT) of rats exposed to AM or LA for 13 weeks (5 days/week for 13 weeks; 6 hr/day) and evaluated at 1 day, 1 month, 3 months, or 18 months after the end of the exposure. Values in legend represent target mass concentrations (mg/m^3^). Results show mean values ± SE (*n* = 4 rats per group). *P* < 0.05 † vs. all other groups; # vs. LA 1.0 group

Development of a fiber dosimetry model will allow more refined analysis of dose, density, dimensions and durability of LA and AM, and exploration of alternative dose metrics. A separate satellite component in this inhalation study was aimed at characterizing fiber burdens in various tissues (nasal cavity, trachea, lung lobes, lymph nodes and pleura) after both acute and chronic exposures to support development of a fiber dosimetry model. The full design strategy for this satellite and associated model development is the subject of other manuscripts. To provide context for the endpoints reported herein, the numbers of all asbestos structures or WHO fibers were determined in the lower respiratory tract (LRT; i.e. five lung lobes) of asbestos-exposed groups at each time point after the 13-week inhalation exposure (Fig. 4). Lung asbestos structures and WHO fibers generally matched patterns of exposure aerosol mass, structure number, and WHO fiber concentrations (Table 2). However, in contrast to the lower aerosol concentration of AM WHO fibers relative to the 3.3 mg/m^3^ LA group, AM lung WHO fibers were comparable to those in the 3.3 mg/m^3^ LA group at 1 day and 1 month post-exposure. Asbestos structures and fibers declined slowly post-exposure. By 3 and 18 months post-exposure, the 3.3 mg/m^3^ LA group, but not the AM group, had significantly greater numbers of lung WHO fibers than the 1.0 mg/m^3^ LA group (Fig. 4). Numbers of lung asbestos structures and WHO fibers were significantly greater in the 10.0 mg/m^3^ LA target group than in all other groups at all times post-exposure. The reduction of LA structures and fibers from the lung over time was generally similar among the 3 LA exposure groups, indicating no impaired clearance of LA fibers at the higher concentrations of LA. Energy-dispersive X-ray spectroscopy (EDS) analysis of lung fibers 18 months post-exposure confirmed the chemistry of the fibers was consistent with LA or AM (Additional file 1: Figure S1). In comparison with the lung, numbers of asbestos structures and WHO fibers were far lower in the upper respiratory tract (URT; i.e. nasal cavity), lung-associated mediastinal lymph nodes, lung pleura, and trachea and larynx (data not shown). Altogether, extra-pulmonary tissue asbestos structures were typically less than 5 % of total (pulmonary + extra-pulmonary) asbestos structures, while extra-pulmonary WHO fibers were always less than 1.5 % of total WHO fibers. Lymphatic clearance of smaller fibers was evident from significantly increased total LA structures in the mediastinal lymph nodes at 1 and 18 months post-exposure in the 10.0 mg/m^3^ LA group compared to all other groups (data not shown). The 10.0 mg/m^3^ LA group also had significantly increased total LA structures in the pleura 1 day after exposure, suggesting greater durability and a possible mechanism for pleural fibrosis (see histopathology results below). Asbestos structures and WHO fibers were sampled in three air control rats 3 months after exposure; 1 to 3 structures were found in all fields from each control rat lung (≤0.2 × 10^4^ calculated total in LRT) and no structures were detected in any other tissues (data not shown).

### Hematology and BALF inflammation

Thirteen weeks of exposure to AM or LA had few significant effects on hematology parameters from 1 day to 18 months after exposure (Additional file 1: Table S1). Compared to air-exposed controls, the percent of lymphocytes among WBCs was significantly reduced 1 day after exposure to AM and 1.0 and 10.0 mg/m^3^ LA, although the absolute number of lymphocytes was not different. No difference from control was observed at later time points. Platelets 1 month after 10.0 mg/m^3^ LA, hemoglobin (Hgb) 3 months after 3.3 mg/m^3^ LA, and mean corpuscular volume (MCV) 3 months after AM were reduced in comparison to air controls, but overall there were no consistent changes in hematology parameters after exposure to AM or LA.

**Fig. 5:**
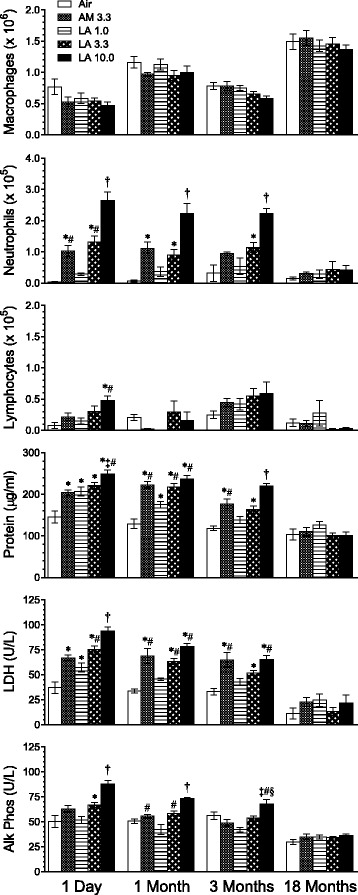
Long-term stop-exposure inhalation study: lung inflammation markers in BALF. Rats were exposed for 13 weeks to AM or LA and evaluated at 1 day, 1, 3, and 18 months post-exposure. Values in legend represent target mass concentrations (mg/m^3^). Results show means ± SE of BALF macrophages, neutrophils, and lymphocytes, and concentrations of protein, LDH, and ALP (Alk Phos) in BALF supernatant (*n* = 8 rats/group). *P* < 0.05 † vs. all other groups; * vs. air control; # vs. LA 1.0; ‡ vs. AM 3.3; § vs. LA 3.3

Compared with air-exposed control groups, numbers of BALF alveolar macrophages were unchanged at all times after exposure to AM or LA (Fig. 5). However, asbestos exposure induces macrophages to become more adherent to airways, likely reducing cell numbers in BALF, whereas macrophage numbers are found to be increased in alveolar duct bifurcations after asbestos exposure [26]. Concentration-dependent increases in BALF neutrophils were observed 1 day, 1 month, and 3 months after AM or LA exposure. Neutrophil numbers were significantly greater in the 10.0 mg/m^3^ LA group compared with all other groups at these times. Neutrophils were similar in AM and 3.3 mg/m^3^ LA groups and significantly greater than air-exposed controls except for AM at 3 months post-exposure. Lymphocyte numbers were increased only in the 10.0 mg/m^3^ LA group 1 day after exposure. BALF supernatant markers of epithelial injury, cytotoxicity, and fibrosis (protein, LDH, and ALP, respectively) were significantly increased from 1 day to 3 months after exposure to AM and higher concentrations of LA (Fig. 5), although group differences were not as marked as with BALF neutrophils. In contrast to results from the range-finding study, no clear differences were observed between the AM and 3.3 mg/m^3^ LA groups. By 18 months post-exposure the neutrophil and lung injury responses had abated and there were no significant differences among groups in BALF parameters. No differences in NAG activity levels were observed at any time post-exposure (data not shown).

### Inflammatory cytokines and Akt and MAPK/ERK pathways

**Fig. 6:**
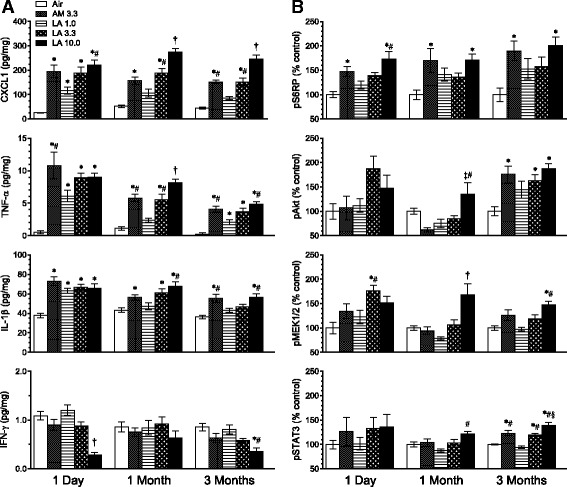
Long-term stop-exposure inhalation study: lung pro-inflammatory cytokines and markers of Akt and ERK pathway activation from right cranial lung lobe tissue. Rats were exposed for 13 weeks to AM or LA and evaluated at 1 day, 1, and 3 months after the end of the exposure. Values in legend represent target mass concentrations (mg/m^3^). Results show mean values ± SE (*n* = 6–7 rats per group). **a** Cytokine levels expressed relative to total protein in sample. **b** Phosphorylated components of Akt pathway (pAkt, pS6RP) and MAPK/ERK cascade (pMEK1/2, pSTAT3) expressed relative to levels in air control group. *P* < 0.05 † vs. all other groups; * vs. air control; # vs. LA 1.0; ‡ vs. AM 3.3; § vs. LA 3.3

Multiple cytokines and chemokines have been demonstrated to play significant roles in particle and fiber-induced pathologies [27, 28]. Chemokine (C-X-C motif) ligand 1 (CXCL1; also known as keratinocyte chemoattractant/growth-related oncogene-alpha (KC/GRO-α)) is a functional homologue of IL-8 and a neutrophil chemoattractant in rodents [29]. TNF-α is a pro-inflammatory cytokine which induces fever, inflammation, and apoptosis [30]. IL-1β is a macrophage-derived mediator of cell proliferation, differentiation, and apoptosis [31]. In lung tissue, all of these cytokines were increased in a concentration-dependent manner by LA from 1 to 3 months after the 13-week exposure (Fig. 6a). Cytokine levels in the AM and 3.3 mg/m^3^ LA groups were comparable. IFN-γ is produced by NK, NKT, and Th1 cells and is critical for innate and adaptive immunity [32]. Starting from a low air control value (~1 pg/mg), IFN-γ was further reduced in the 10.0 mg/m^3^ LA group compared to all other groups at 1 day, and compared to air control and 1.0 mg/m^3^ LA at 3 months after exposure. Although IL-13 contributes to pulmonary fibrosis [33], lung tissue levels of pro-allergic cytokines such as IL-4 and IL-13 were very low in all groups (<0.5 pg/mg), and IL-5 did not have consistent time- or concentration-dependent responses (data not shown).

The Akt (also known as protein kinase B) signaling pathway regulates diverse cellular functions including metabolism, growth, proliferation, cell survival, transcription and protein synthesis [34]. In lung tissue, levels of activated pAkt (phospho-Akt (Ser473)) were significantly different from control levels in the AM and 3.3 mg/m^3^ and 10.0 mg/m^3^ LA groups only at 3 months after exposure (Fig. 6b). In contrast, pS6RP (phospho-S6 ribosomal protein (Ser240/244)), activated downstream of pAkt or by an alternative pathway [35], was elevated in the 10.0 mg/m^3^ LA group at all times from 1 day to 3 months post-exposure. pS6RP was also elevated in the 3.3 mg/m^3^ AM group but not the corresponding 3.3 mg/m^3^ LA group at all time points. Levels of p70S6K (phospho-70 kDa ribosomal protein S6 kinase (Thr389)) and pGSK-3β (phospho-glycogen synthase kinase-3β (Ser9)) were not different from control levels at any time after exposure (data not shown). Components of the MAPK/ERK pathway regulate functions including proliferation, differentiation, and development of malignancies [36, 37]. Levels of activated pMEK1/2 (phospho-mitogen-activated protein ERK kinase 1, 2 (Ser217/221)) were increased over control levels in the 10.0 mg/m^3^ LA group at 1 month and 3 months post-exposure, and at 1 day after exposure in the 3.3 mg/m^3^ LA group (Fig. 6b). Levels of pERK1/2 (phospho-extracellular signal-regulated kinase 1, 2 (Thr202/Thr204; Thr185/Tyr187)), downstream from pMEK1/2 [37], were not significantly different from control levels at any time (data not shown). Levels of activated pSTAT3 (phospho-signal transducer and activator of transcription 3 (Tyr705)), which may be activated by Janus kinases in a separate pathway from MEK1/2 [38], were increased over control levels in the AM and 3.3 mg/m^3^ and 10.0 mg/m^3^ LA groups only at 3 months after exposure.

### Survival and histopathology

Rats which survived 18 months post-exposure were 100 weeks old at the scheduled necropsy. Exposure to AM and varying concentrations of LA for 13 weeks did not significantly affect early mortality before scheduled necropsy, which ranged from 9 to 19 rats in each group of 50 (18–38 %). Average lifespan was 97.7, 98.0, 96.4, 98.2, and 97.8 weeks for 18-month control, AM, and 1.0, 3.3, and 10.0 mg/m^3^ LA groups, respectively (*P* = 0.42) (Additional file 1: Figure S2). The leading cause of mortality among early deaths was mononuclear cell leukemia (MCL), which is a common finding in older male Fischer rats [39, 40] (Additional file 1: Table S2). No significant group differences were observed for the incidence of MCL or other findings in early death animals, and rates were comparable to earlier studies [33].

**Fig. 7:**
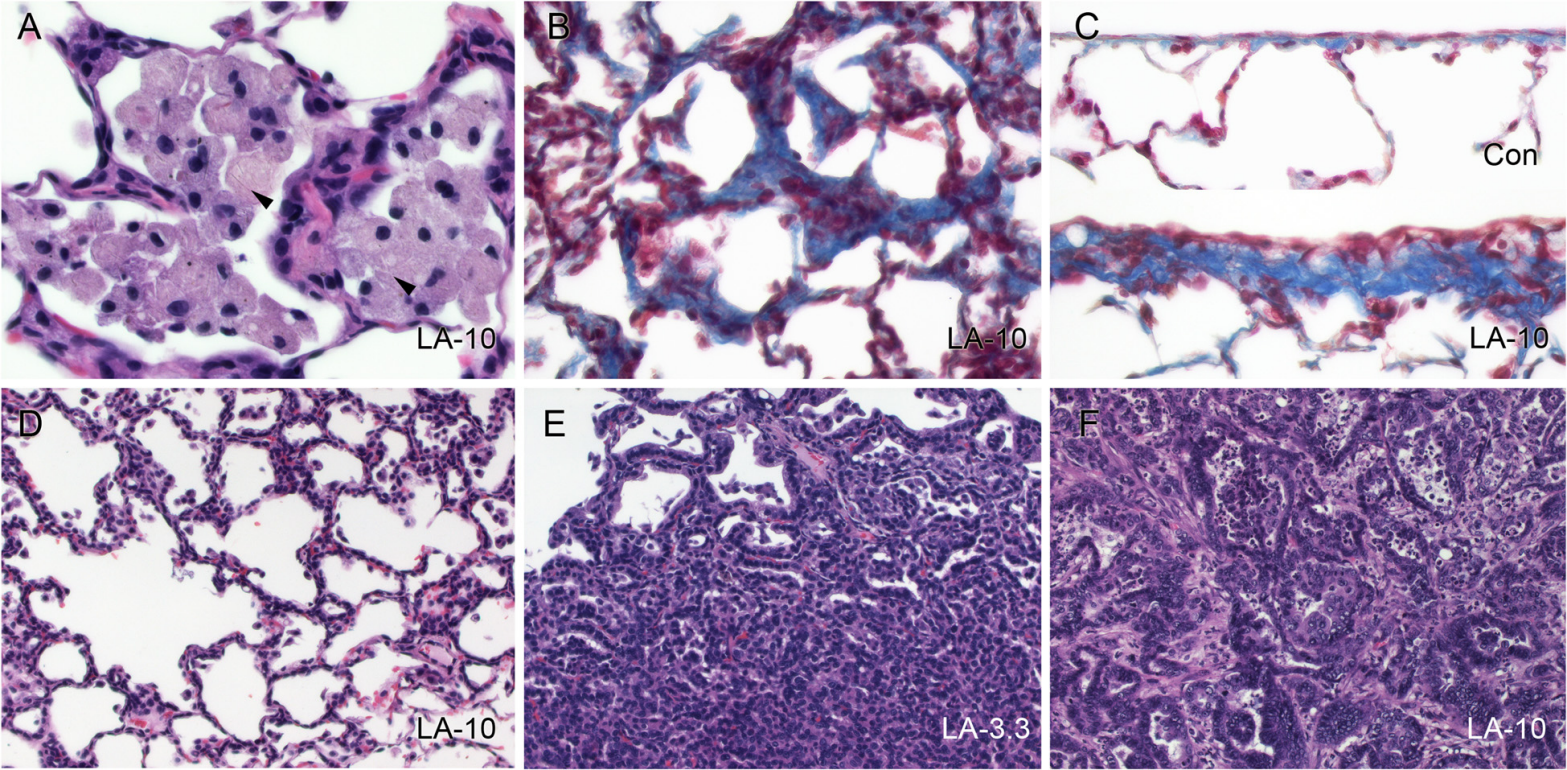
Stop-exposure inhalation study: persistent effects on histopathology 18 months after exposure to AM or LA for 13 weeks. All images shown are from 10 mg/m^3^ LA target mass concentration except panel E (3.3 mg/m^3^ LA). Images represent **a** alveolar inflammation (macrophages) with intracytoplasmic fibers (*arrowheads*), **b** interstitial fibrosis (indicated by blue-stained collagen), **c** subpleural fibrosis (*lower image*) compared to normal pleura (*upper image*), **d** alveolar epithelial hyperplasia (indicated by cuboidal type II pneumocytes lining alveolar walls), **e** bronchiolar/alveolar (B/A) adenoma, and **f** B/A carcinoma. Slides were stained with either hematoxylin and eosin (**a**, **d**–**f**) or Masson’s trichrome (**b**, **c**). Images were taken at 60x (**a**), 40x (**b**, **c**), or 20x (**d**–**f**) objective magnification

Histopathological findings 1 day post-exposure in the 13-week study included foreign bodies (consistent with fibers) within alveolar macrophages, alveolar inflammation, interstitial fibrosis, and bronchiolization in all fiber-exposed groups and bronchiolar epithelial hyperplasia only in the 10.0 mg/m^3^ LA group (Table 3). The abundant alveolar macrophages are consistent with prior studies showing these cells as the predominant inflammatory cell type in alveoli following asbestos exposure [41]. Granulomas composed of macrophage aggregates and rare giant cells were occasionally present, consistent with a foreign body response. Similar findings were evident in all AM and LA groups 1 and 3 months post-exposure, with the exception of bronchiolar epithelial hyperplasia at the 3-month time point. At the 18-month recovery period, scant fibers could still be identified within macrophages and scattered multinucleated giant cells in the majority of the animals from each fiber group (Fig. 7a). Fibers did not accumulate to the degree associated with overload of alveolar macrophages, indicating that overload did not contribute to the observed pathological changes. Other histopathological findings in all fiber-exposed groups included alveolar inflammation and interstitial, pleural, and subpleural fibrosis (Fig. 7b, c). The AM and 10.0 mg/m^3^ LA groups also had a higher incidence of alveolar epithelial hyperplasia (Fig. 7d) which showed a positive dose trend among LA groups (*P* = 0.0099).

**Table 3:** Lung and trachea histopathology after exposure to AM or LA for 13 weeks

	Air control	AM 3.3	LA 1.0	LA 3.3	LA 10.0
*Lung*					
Foreign Body^a^					
1 Day	0	8*	8*	8*	8*
1 Month	0	8*	8*	8*	8*
3 Months	0	8*	8*	8*	8*
18 Months	0	47*	40*	48*	49*
Alveolus Inflammation^b^					
1 Day	0	8 (1.0)*	8 (1.0)*	8 (1.0)*	8 (2.0)*
1 Month	0	8 (1.1)*	8 (1.0)*	8 (1.0)*	8 (2.0)*
3 Months	0	8 (1.0)*	8 (1.0)*	8 (1.1)*	8 (1.4)*
18 Months	0	45 (1.0)*	40 (0.9)*	47 (1.1)*	48 (1.1)*
Alveolar Epithelial Hyperplasia^c^					
1 Day	0	0	0	0	0
1 Month	0	0	0	0	1 (0.1)
3 Months	0	0	0	0	1 (0.1)
18 Months	1 (0.0)	9 (0.3)*	6 (0.2)	6 (0.2)	9 (0.4)*^
Bronchiole Epithelial Hyperplasia					
1 Day	0	0	0	0	8 (1.0)*
1 Month	0	1 (0.1)	0	0	8 (1.0)*
3 Months	0	0	0	0	0
18 Months	0	0	0	0	0
Bronchiolization^d^					
1 Day	0	8 (1.0)*	7 (0.9)*	8 (1.0)*	8 (1.0)*
1 Month	0	7 (0.9)*	7 (0.9)*	7 (0.9)*	8 (1.4)*
3 Months	0	8 (1.0)*	8 (1.0)*	8 (1.0)*	8 (1.0)*
18 Months	0	5 (0.1)	5 (0.1)	0	0
Interstitial Fibrosis^e^					
1 Day	0	8 (1.0)*	8 (1.0)*	8 (1.0)*	8 (1.0)*
1 Month	0	8 (1.0)*	7 (0.9)*	8 (1.0)*	8 (1.0)*
3 Months	0	8 (1.0)*	8 (1.0)*	8 (1.0)*	8 (1.0)*
18 Months	0	42 (0.8)*	29 (0.6)*	43 (0.9)*	42 (0.9)*
(Sub) Pleural Fibrosis^e^					
18 Months	0	25 (0.5)*	16 (0.3)*	19 (0.4)*	22 (0.4)*
Bronchiolar/Alveolar Adenoma					
18 Months	0	2	0	2	3^
Bronchiolar/Alveolar Carcinoma					
18 Months	0	0	0	0	1
Bronchiolar/Alveolar Adenoma or Carcinoma					
18 Months	0	2	0	2	4^
*Trachea*					
Trachea, Chronic Inflammation					
18 Months	0	0	4 (0.1)	2 (0.1)	9 (0.2)*
Trachea, Squamous Metaplasia					
18 Months	0	1 (0.0)	0	0	0

Rats were exposed to AM or LA for 13 weeks and evaluated at 1 day, 1 month, 3 months, or 18 months after the end of the exposure period. Column headings indicated target mass concentrations (mg/m^3^). Values represent the number of animals with a finding in each group (total examined *n* = 8 at 1 day, 1, and 3 months, and *n* = 49–50 at 18 months). Average lesion severity scores for all rats in each group (shown in parentheses) were graded as follows: 1 = minimal, 2 = mild, 3 = moderate, 4 = marked, and 5 = severe. *P < 0.05 vs. air control; ^*P* < 0.05 for trend test among LA groups. ^a^Consistent with fibers; not graded for severity. ^b^Predominantly macrophages with lesser numbers of neutrophils and lymphocytes, often associated with foreign bodies; occasional granulomas with rare giant cells. ^c^Proliferation of cuboidal type II alveolar cells (without evidence of significant inflammation). ^d^Presence of cells that resemble bronchiolar epithelial cells in alveolar ducts and adjacent alveoli. ^e^Confirmed on Masson’s trichrome stain

Bronchiolar/alveolar (B/A) tumors (adenoma and carcinoma) were observed in 8 animals (2 each in the AM and 3.3 mg/m^3^ LA groups (4 % incidence) and 4 in the 10.0 mg/m^3^ LA dose group (8 % incidence)) (Table 3). Microscopically, B/A adenomas presented as discrete masses composed of ribbons and sheets of cuboidal cells that filled alveolar spaces and compressed adjacent tissue (Fig. 7e). Neoplastic cells within adenomas were typically monomorphic with low rates of mitotic activity. The one carcinoma exhibited cytologic atypia, local and vascular invasion (Fig. 7f), and metastasis to the thoracic cavity, diaphragm, kidney, ribs, sternum, and heart leading to death. There was no significant pairwise difference in the incidence of lung adenoma and/or carcinoma between groups (unadjusted *P* = 0.058 using time-adjusted analysis). However, there was a statistically significant trend in tumor incidence for combined pulmonary adenoma or carcinoma using a Cochran-Armitage Trend Test (Exact Test) (*P* = 0.0064 for all 18-month animals with pathology data; *P* = 0.0067 using time-adjusted analysis). No pleural mesotheliomas were observed in any of the treatment groups.

In systemic tissues examined other than the lung, there was greater incidence of minimal to mild chronic tracheal inflammation only in the 10.0 mg/m^3^ LA group (Table 3). This change was characterized by an infiltration of mononuclear cells in the tracheal submucosa. Other spontaneous/background changes included hypospermia in the epididymal tubules and tubular atrophy, edema, and interstitial cell adenomas in the testis (Additional file 1: Table S3). The incidence and severity of these findings in the fiber-exposed groups were similar to the air control group. All other histopathological findings were considered incidental.

## Discussion

This study was designed to evaluate internal dose, time course dynamics of pathological effects, and potential target pathways for inhaled LA relative to AM, a distinct amphibole with established toxicity. Lung fiber burdens after 13-week exposures were concordant with aerosol mass, asbestos structure number, and WHO fiber concentrations, and were similar between matched concentrations of LA and AM. Asbestos structure and fiber burdens declined gradually post-exposure but approximately half the original lung burden was still present 18 months after exposure. The biological potency of inhaled LA in the 13-week study was comparable to that of AM on a mass basis. After short-term exposures, some of the early inflammatory responses such as LDH, protein, pro-inflammatory cytokine expression, and alveolar inflammation were more severe for LA compared to the same mass concentration of AM, but following the 13-week study, early and persistent effects of LA and AM were similar in terms of inflammation markers and respiratory tract pathology. Several target pathways implicated in tumorigenesis were observed after asbestos exposure, including Akt, MAPK/ERK, and pro-inflammatory cytokines. These findings clearly demonstrate persistence of inflammatory and fibrotic responses to relatively short LA fibers which may drive proliferative effects and contribute to lung tumorigenesis long after primary exposure.

Several reviews and assessments have evaluated the risk of respiratory tract cancer among vermiculite workers at Libby [1, 3, 6, 11, 42]. These studies consistently reported a higher overall risk of death due to lung or other respiratory tract cancers, with mortality rates from 1.4 to 2.4 times higher than control non-exposed populations. Exposure-response analyses from these studies further showed a positive relationship between lung cancer mortality and exposure, using different measures of exposure, lag periods, and exposure metrics, with ~2- to 3-fold greater risk in the highest exposure group [1]. In this study a dose trend for increased lung tumors was observed among the LA-exposed groups. This effect is considered to be treatment-related given the statistical significance (*P* < 0.01), the time- and dose-concordance of related early effects (e.g. increased cell proliferation, hyperplasia, inflammation), and supporting data from human epidemiologic studies [1]. Lung tumor incidence for AM and 3.3 mg/m^3^ LA groups (4 %) was similar to historical control estimates previously reported in aged male F344 rats. For example, at the National Toxicology Program (NTP), the historical control incidence of combined B/A tumors in aged male Fischer 344 rats is ~3.6 % [39]. However, historical control values may vary by site/institution and sectioning protocol, so direct comparisons should be treated with caution. Only left lung was examined on histopathology here, resulting in a lower sampling intensity compared to other protocols in which both left and right lung lobes are examined [43].

The MOA for lung tumorigenicity in response to LA has not been clearly established. Proposed key events include sustained local cytotoxicity and inflammation leading to increased cellular proliferation and hyperplasia [1]. Findings from this study support this MOA, showing that LA induces these events as direct effects which then persist long after exposure has ended. Cytotoxicity was indicated in the short-term study by increased LDH in BALF and inflammation with pyknotic nuclei and karyorrhectic debris in all fiber-exposed groups at all time points on histopathology. Inflammation was also indicated by BALF markers in higher-dose LA groups and gene markers in mid- and high-dose LA groups. Bronchiolar epithelial cell proliferation was significantly higher in the 3.3 and 10.0 mg/m^3^ LA groups and showed a clear dose trend among LA groups. This finding was supported by higher incidence of bronchiolar epithelial hyperplasia following 10 days exposure (25 mg/m^3^ LA) and 1 day and 1 month following 13 weeks of exposure (10 mg/m^3^ LA), and alveolar epithelial hyperplasia at the 10 mg/m^3^ LA dose 18 months after the 13-week exposure. Increased BrdU LI in TB epithelial cells was found to be the best predictive measure of long-term pathogenicity of fibers among several short-term in vitro and in vivo endpoints [24]. Inflammation and histopathology findings observed in this study were comparable to a recent study in which male F344 rats were exposed to LA or AM by intratracheal (IT) administration, with fibers given in single or multiple bolus doses over a 13-week period [44]. In that study, LA produced greater acute inflammation and lung injury than AM, but long-term histopathological changes were generally equivalent, and similar to effects observed in the present study with 10-day and 13-week exposures. Low numbers of B/A adenomas, B/A carcinomas, and mesotheliomas were found 20 months after IT exposure to AM or LA [44], but with 24 animals per exposure group, no significant dose trend could be detected.

This study found that in comparison to air control, inhalation exposure of rats for 10 days to 3.5 mg/m^3^ AM or LA up to 25 mg/m^3^ did not change mRNA expression of inflammasome pathway components, including *Nlrp3*, *Pycard*, and *Casp1*, and only slightly increased expression of genes for the downstream cytokines *Il1b* and *Il18*. IL-1β production is typically mediated by inflammasome-dependent caspase activation, although other proteases can also process precursor IL-1β to its active form (as demonstrated in studies of diesel exhaust particulate exposure which induced pulmonary inflammation and IL-1β in NLRP3 and caspase-1 knockout mice [45]). Several studies utilizing high dose-rate exposure techniques (IT, intranasal (IN), and in vitro) have implicated the inflammasome pathway in the development of asbestos- and particle-induced pathology. Lung expression of *Nlrp3* (Nalp3), *Pycard* (ASC), *Il1b*, and *Ctsb* (cathepsin B, an activator of the inflammasome pathway) were increased 4 h after IT instillation of rats with 0.5 mg LA [46]. In comparison to wild-type mice, lung inflammation and collagen deposition were significantly reduced in knockout mice deficient in ASC or Nalp3 3 months after IN instillation of 2 mg silica [21]. Finally, high asbestos and silica concentrations in vitro stimulated macrophage IL-1β production, which was dependent on the presence of inflammasome components and reactive oxygen species production [22]. Although inhalation exposure for 8 days to chrysotile asbestos (7 mg/m^3^) was reported to produce lower BALF inflammation and pro-inflammatory cytokines in *Nlrp3* knockout mice compared to wild type mice, the effects shown were marginal [22]. Together, these data suggest that asbestos or silica exposure achieved through high dose-rate bolus IT, IN, or in vitro exposures may activate the inflammasome pathway, but more physiologically relevant inhalation exposures to AM or LA as in the present study do not stimulate this pathway to a significant degree. Differences in the animal models used in studies examining inflammasome activation may also play a role in the variability of the response.

Chronic inflammation may play an important role in the development of neoplasia [17, 18]. Although we did not find evidence for activation of the inflammasome pathway, other mediators of inflammation were elevated after AM or LA exposure. Macrophages produce CXCL1 and CXCL2 which bind to C-X-C chemokine receptor 2 (CXCR2), induce neutrophil recruitment [47], and mediate growth factor signaling networks that may contribute to carcinogenesis [48]. Expression of *Il6*, *Tnfa*, and *Cxcl2* were increased in a dose-dependent manner following the 10-day exposure. TNF-α as well as CXCL2 are potent neutrophil recruitment factors, which may contribute to the persistent neutrophilic response in the lung up to 3 months following the 13-week exposure. As with measures of BALF protein and LDH, exposure to 3.5 mg/m^3^ LA, but not AM, significantly increased lung tissue *Tnfa* and *Cxcl2*, suggesting that LA was more potent than AM in producing an acute inflammatory response leading to lung injury. Following the 13-week exposure, lung tissue protein levels of CXCL1, TNF-α, and IL-1β were increased in AM- and LA-exposed groups. In contrast to the 10-day exposure, responses following AM were comparable to those of LA at the same exposure level, suggesting that persistent inflammatory responses to subchronic AM or LA exposure were similar. Our results are consistent with previous studies showing increases in expression of *Cxcl1* and *Cxcl2* in lung tissue after IT exposure of rats to particles or inhalation exposure of mice to crocidolite asbestos [49]. These results suggest that cytokine-mediated neutrophil inflammation may have an important role in the progression of lung injury and activation of growth factor pathways involved in tumor formation. Furthermore, TNF-α also plays a critical role in asbestos-induced interstitial fibrosis and cellular proliferation, as demonstrated in TNF-α receptor knockout mice which had no increase in BrdU labeling or fibrotic lesions after chrysotile exposure [50]. These effects of TNF-α may in turn be mediated through activation of transforming growth factor-β1 [51].

The Akt pathway is activated through membrane-bound receptor tyrosine kinases and phosphatidylinositol 3-kinase (PI3K), resulting in phosphorylation of Akt on Ser473, among other sites [52]. pAkt promotes cell survival by inactivation of pro-apoptoptic proteins, including GSK-3β by phosphorylation at Ser9 [34]. This study found pAkt in lung tissue was elevated above control levels 3 months after the 13-week exposure to AM or LA, while downstream pGSK-3β (Ser9) was unchanged from control levels from 1 day to 3 months post-exposure, suggesting that any inhibitory effects of AM or LA on apoptosis occur by other pathways. Activated pS6RP, which has a significant role in control of cell size and growth [35, 53], may occur downstream from pAkt or separately through activation by phosphoinositide-dependent kinase 1 (PDK1) [35]. Further work is needed to define the possible role of pS6RP in mediating effects of AM and LA on bronchiolization and alveolar epithelial hyperplasia. Active p70S6K (Thr389), one of the kinases which phosphorylate S6RP [35, 53], was not elevated above control levels at any time. Overall, these results suggest a limited role for components of the Akt pathway in events leading to tumor development following exposure to AM or LA.

Previous studies have shown that the MAPK/ERK pathway can play an important role in the development of inflammation leading to tumor development after asbestos, oxidant, or cigarette smoke exposure [54]. Increased levels of pMEK1/2 were found in lung tissue after the 13-week exposure to higher concentrations of LA, although levels of pERK1/2, downstream of pMEK1/2 [37], were not consistently elevated. pSTAT3 may be activated downstream of pMEK1/2 or separately by Janus kinases [37, 38], and elevated levels of pSTAT3 were found 3 months post-exposure to AM or LA. Since this transcription factor promotes cell cycle progression, cell transformation, and prevention of apoptosis [38], elevation of pSTAT3 may also contribute to cell growth signaling associated with tumor promotion.

The acute phase response is an essential component of the innate immune system which is activated by infection, stress, and other stimuli [55]. Following IT dosing of WKY or F344 rats with LA, several acute phase response proteins were increased in serum, suggesting a role for these markers in mediating the systemic inflammatory response to fibers [56]. The effects of AM or LA delivered by inhalation or IT dosing on serum markers were compared recently in samples from a comparative toxicity study [57] and the present inhalation study [58]. One day after IT dosing of LA (0.5 or 1.5 mg/rat), the acute phase marker α-2 macroglobulin was increased more than 10-fold compared with 1 day after the 13-week exposure to 10 mg/m^3^ LA, but no other acute phase response or metabolic impairment markers were increased after inhalation exposure. Other mediators of inflammatory (and potential tumorigenic) responses to LA include osteopontin and mesothelin [59, 60]. Serum osteopontin was increased 1 day after IT LA in WKY rats [56], and mesothelin was increased in lung tissue 2 years after IT LA in F344 rats [61]. Eighteen months after the 13-week exposure to 10.0 mg/m^3^ LA in rats from the present study, both osteopontin and mesothelin were increased (though not significantly) in serum [58]. These studies indicate that exposure to LA may initiate systemic immune and inflammatory responses which interact with local effects in the lung.

The toxicity of asbestos or asbestiform minerals has often been attributed to their fiber characteristics. Although it is probable that shorter non-asbestiform (prismatic or acicular) fibers are less hazardous than longer asbestiform fibers [62], all naturally occurring asbestos samples contain a wide distribution of sizes, making it critical to thoroughly analyze the physical characteristics of the samples being tested. We found that LA WHO fibers were more persistent in the lung than AM WHO fibers, suggesting a greater potential for long-term toxicity with LA exposure. However, density and inhaled size distribution influence aerodynamics and initial deposition patterns, while the durability of fibers in vivo influences clearance mechanisms. Caution in assigning relative toxicity of fiber types is prudent until further elucidation of the dose-response relevant to different endpoints can be more fully explored. Evaluation of additional dose metrics such as fiber number or surface area with various normalizing factors such as epithelial surface area or number of alveolar macrophages may provide additional useful insights on MOA. Dosimetry models can predict various internal dose metrics to inform the MOA for amphibole fibers, address interspecies extrapolation, characterize uncertainty in exposure-response models, and help to integrate evidence across different experimental designs [63].

## Conclusions

This study showed that inhalation of LA in rats produces inflammatory, fibrogenic, and tumorigenic effects in the lung which include key features of asbestos-associated disease in humans. Long-term effects were produced by subchronic exposures to samples that contained predominantly shorter fibers. Short-term effects included lung inflammation, terminal bronchiolar epithelial cell proliferation, and pro-inflammatory cytokine expression. Tissue fiber burdens persisted up to 18 months following subchronic inhalation, emphasizing the important role of fiber dosimetry in determining long-term (post-exposure) effects on the respiratory tract. Markers of lung injury and pro-inflammatory cytokines persisted up to 3 months post-exposure, while inflammatory and fibrotic changes on histopathology persisted up to 18 months. Significant trends for alveolar epithelial hyperplasia and B/A lung tumors were observed 18 months after exposure among LA groups, consistent with short-term effects on cell proliferation. Examination of several inflammatory pathways involved in tumorigenesis found no support for a role of the inflammasome pathway, limited support for contributions of the Akt and MAPK/ERK pathways, and the strongest support for the function of persistent pro-inflammatory cytokines in mediating long-term LA effects. Collectively, this evidence indicates that inflammation and activation of growth factor pathways may persist and contribute to lung tumorigenesis long after initial LA exposure. These findings should contribute to risk assessment of LA and the development of dosimetry models of amphibole fibers.

## Methods

### Asbestos samples

Libby amphibole samples were collected from the Rainy Creek Complex located near Libby, Montana, in 2007 and processed by the U.S. Geological Survey (Denver, CO) into finer materials as described previously [64]. Union for International Cancer Control (UICC) AM was used as a reference sample (CAS No. 12172-73-5). Sample characteristics of LA and AM were previously reported (Table 1 of [65] and associated online Additional file 1).

### Experimental design

A 2-week range-finding inhalation study was conducted to guide exposure levels for the subsequent 13-week inhalation study. Five groups of rats were exposed nose-only 5 days/week for 6 h/day (10 days total) to high-efficiency particulate air (HEPA)-filtered air only, LA aerosol spanning a 50-fold range (0.5, 3.5, and 25.0 mg/m^3^), or AM at 3.5 mg/m^3^. Rats were necropsied immediately after the final exposure for analyses of lung inflammation and gene expression (*n* = 7/group), or 4 days after final exposure for assessment of lung histopathology and bronchiolar epithelial cell proliferation (*n* = 7/group). For the 13-week inhalation study, five groups of rats were exposed nose-only for 6 h/day 5 days/week to HEPA-filtered air only, LA aerosol spanning a 10-fold range (1.0, 3.3, and 10.0 mg/m^3^), or AM at 3.3 mg/m^3^. Rats were necropsied 1 day, 1, 3, and 18 months after the last exposure day (*n* = 8/group at 1 day, 1 month, and 3 months post-exposure, and *n* = 50/group at 18 months post-exposure). Additional groups of rats (*n* = 3–6/group) were exposed to determine fiber burdens in the URT, trachea/larynx, LRT, pleura, and mediastinal lymph nodes. For all time points, BALF was collected from right lung lobes in eight animals/dose group for cellular and lung injury endpoints, and left lungs were collected for histopathology. At 1 day, 1, and 3 months post-exposure, right cranial lung lobes were assessed for inflammatory cytokines and proteins related to cell growth, proliferation, and survival pathways. Animals in the 18-month post-exposure arm were assessed for gross changes and histopathology of left lung lobe, trachea, sternum, pleura, testis, epididymis, and any gross lesions.

### Exposure system

Five direct-flow nose-only exposure systems (RCC, Geneva, Switzerland) were used in the study: 3 for the LA aerosol, 1 for the AM aerosol, and 1 for the air control. The exposure systems consisted of modular 16-port tiers and were each configured into 6- or 7-tier towers located in separate 8 m^3^ chambers for exposure containment and personnel safety (Additional file 1: Figure S3). HEPA-filtered and conditioned supply and exhaust fans controlled air flow through the 8 m^3^ chambers, and the exhaust from each chamber was HEPA-filtered. Animals were placed in open nose-only exposure restraint tubes (Battelle Memorial, Richland, WA) and attached to the towers via tier ports. For the 13-week inhalation study, airflow into the towers was maintained at 50 L/min (air control) or 57 L/min (LA and AM exposures), and exhaled air and excess exposure airflow were delivered into the tower exhaust plenum. Each 8 m^3^ chamber was maintained at an average of 20–22 °C and 48–52 % relative humidity, and each tower was kept at 22–26 °C and 5–9 % relative humidity, representing animal environment and inhaled atmosphere conditions, respectively. Four rotating brush aerosol generators (models CR-3000 or CR-3020, CR Equipements SA, Tannay, Switzerland) were used to gently loft and aerosolize the fiber test material for the LA and AM atmospheres. Each generator controlled movement of a piston gently packed with a column of sample into a rotating brush, which swept material off the top of the column into the HEPA-filtered air stream. Generator brush and piston speeds were adjusted to achieve required aerosol mass concentrations. Sample fibers were delivered past a Kr^85^ source (10 mCi; Isotopes Products Inc., Valencia, CA) to reduce particle charges.

### Exposure characterization

To determine aerosol concentration stability during exposures, a light scatter respirable aerosol monitor (RAM; MIE Inc., Billerica, MA) sampled aerosol continuously from a port at the tower inlet. Mass concentrations in each tower were measured daily using glass fiber filters (Pall Life Sciences, Ann Arbor, MI) collected from an exposure port and weighed on a microbalance (ATI CAHN model C31, Boston, MA). Particle size distributions were determined weekly using a calibrated optical particle sizing spectrometer (Aerodynamic Particle Sizer Model 3321, TSI, Inc., St. Paul, MN) connected to a port at the tower inlet, avoiding overload by dilution with HEPA-filtered air as necessary. Fiber size distributions were determined weekly on samples collected on polycarbonate filters. These filters were adhered to 25 mm aluminum pin mounts with conductive lubricant (Neolube No.20, Huron, IN) and gold-coated (210–280 Å thickness) with a sputter coater (SPI-Module model 12151, West Chester, PA). An SEM (JEOL model JSM-840A, Tokyo, Japan) captured sample images from the gold-coated filters, which were analyzed with Image-Pro Plus (v5.0.1.11 for Windows/XP, Media Cybernetics Inc., Bethesda, MD) to count the number of all structures with any dimension ≥0.2 μm and measurements of associated L and D across 15 fields at 5000x magnification. Sample mass and size distributions measured at top, middle, and bottom ports of each tower prior to the start of animal exposures showed the samples were uniformly distributed throughout the exposure towers.

### Animals

Animal use protocols were approved by the Institutional Animal Care and Use Committees of the U.S. EPA and the Hamner Institutes for Health Sciences and followed in accordance with the Guiding Principles in the Use of Animals in Toxicology and all relevant Animal Welfare Act regulations in effect at the start of this study. Male F344 (CDF) rats (Charles River Laboratories, Raleigh, NC) were housed a minimum of 10 days before the start of the studies in an AAALAC-accredited, specific-pathogen-free facility under standard conditions (20–24 °C, 30–70 % relative humidity, 12/12 h light/dark cycle). Rats were housed in individual wire mesh cages in the 8 m^3^ chambers during the 10-day and 13-week exposure periods and in solid bottom cages with bedding in separate rooms after the exposure periods. At the beginning of the 13-week inhalation exposure, rats were 10 weeks old and weighed 200 ± 15 g (mean ± SD). All animals received Certified Rodent Diet NIH-07 pellets (Zeigler Bros., Gardners, PA) and filtered water *ad libitum*. Animal body weights were recorded every week and health status was monitored daily.

### Tissue fiber burden

Tissue fiber burdens were determined 1 day, 1, 3, and 18 months after the 13-week exposure period. Animals were euthanized with intraperitoneal sodium pentobarbital (up to 200 mg/kg) and exsanguinated via the abdominal aorta. The head was removed and the URT was isolated and stored at -80 °C. Aqueous 2 % agarose (type 1-B; Sigma-Aldrich, St. Louis, MO) with 0.1 % SDS (Sigma) heated to 45 °C was injected slowly into the pleural cavity, then heated agarose without SDS was instilled into the lungs via a tracheal cannula [66]. After placement of the carcass on ice for 30 min, the trachea and larynx, LRT, pleural tissue, and mediastinal lymph nodes were removed and stored at -80 °C until analyzed. Samples were weighed before and after 6 h of freeze-drying (Freezone system, Labconco, Kansas City, MO) and then again following overnight freeze-drying to ensure complete dryness. Freeze-dried samples were weighed before low-temperature plasma ashing (LTA-504, LFE Corporation, Springfield, VA) and 24 h later, then ashed up to 96 h to ensure stable weight and complete ashing. Ashed samples were resuspended in water and spotted on polycarbonate filters, processed, and analyzed as described above for the aerosol samples to obtain numbers of fibers and their associated dimensions (L, D). EDS was conducted on a JEOL JSM6510LV SEM equipped with an EDAX Genesis XM2 EDS analyzer system on a minimum of five structures per sample from rats 18 months post-exposure to AM or LA to confirm fiber identity.

### Complete blood cell and bronchoalveolar lavage fluid (BALF) analyses

Animals were anesthetized and exsanguinated as described for fiber burden analysis, and blood was collected in ethylenediaminetetraacetate (EDTA)-coated tubes for complete blood cell analysis. Blood was analyzed for hemoglobin (Hgb), hematocrit (Hct), mean corpuscular (MC) volume, MC Hgb (MCH), MC Hgb concentration (MCHC), and numbers of white blood cells (WBC), red blood cells (RBC), lymphocytes, and platelets using a hematology analyzer (AcT 10; Beckman Coulter, Miami, FL). The left lobe was ligated and right lung lobes were lavaged with 3 ml PBS 5 times, keeping the first lavage separate. BALF was centrifuged at 200 x g for 10 min, and combined cell pellets were resuspended in 1 ml F12 medium and counted using a ZM Coulter Counter (Beckman Coulter, Brea, CA). BALF cell slide preparations were stained with Diff-Quick (Fisher Scientific, Kalamazoo, MI), and 300 cells per sample were differentiated as macrophages, neutrophils, or lymphocytes. The first lavage supernatant was analyzed for markers of lung injury, including protein content and LDH, ALP, and NAG activities, using standard reagents and protocols (Thermo Scientific, Rockford, IL; Carolina Liquid Chemistries, Brea, CA; Pointe Scientific, Canton, MI; and Roche Diagnostics, Indianapolis, IN). All assays were conducted on a Cobas Fara II (Roche Diagnostics) or AU600 (Olympus America, Center Valley, PA) clinical chemistry analyzer.

### Histopathology

Necropsy procedures were conducted by pathology staff from Experimental Pathology Laboratories, Inc. (EPL®, Morrisville, NC) at The Hamner Institute for Health Sciences (Research Triangle Park, NC). In rats selected for BALF, right lung lobes were removed after fluid collection, frozen in liquid nitrogen, and stored at -80 °C for analysis of RNA or protein markers as described below. The left lung lobe ligature was removed, and the trachea and left lung lobe were instilled and fixed *in situ* with 10 % neutral buffered formalin (NBF) at approximately 30 cm NBF pressure. Left lung, trachea, sternum (including pleura), left testis and epididymis, and any potential lesions observed on gross examination were fixed with NBF for 48 h, rinsed, and stored in 70 % ethanol. Two early deaths in the 18-month post-exposure arm (one each in the low-dose and mid-dose LA groups) were not evaluated on histopathology due to tissue autolysis.

Fixed tissues were embedded in paraffin wax, sectioned at approximately 5 μm, deparaffinized, and stained with hematoxylin and eosin (H&E) at EPL. Additional slides from the left lung, trachea, and sternum were stained with Masson-Goldner trichrome stain to evaluate collagen content (as an indicator of interstitial or (sub) pleural fibrosis). H&E- and trichrome-stained sections were evaluated via light microscopy by an experienced board-certified pathologist at EPL (GAW). Histopathologic examination used standard criteria and nomenclature for neoplastic and non-neoplastic lesions [40, 67, 68]. Select microscopic findings were graded using a subjective grading scale (1 = minimal, 2 = slight/mild, 3 = moderate, 4 = moderately severe, 5 = severe). Mean severity grades were calculated by dividing the sum of the severity grades for a finding in a group by the number of animals examined. Findings from early death animals were included with originally assigned groups when pathology data were available. Gross findings at necropsy were correlated with histological findings when possible. Slides were imaged using an Infinity2 digital camera (Lumenera, Ottawa, ON).

### Terminal bronchiolar epithelial cell proliferation

One day after the 10-day exposure period, seven rats/group were surgically implanted in the dorsal thoracic region with micro-osmotic pumps (Model 2 ML1 7-day pump; Alzet, Cupertino, CA) using aseptic procedures under isoflurane anesthesia. The pumps were filled with BrdU (5 mg/mL in PBS), which was administered at 10 μL/h for 3 days. BrdU is a thymidine analog that selectively labels cells entering the S-phase of the cell cycle. Four days after the exposure period, animals were necropsied and the left lung lobe from each animal was fixed in NBF, transferred to ethanol, and processed as described above. Lung sections were stained for BrdU by EPL using standard immunohistochemical procedures which included a primary mouse monoclonal antibody for BrdU diluted 1:50 (Clone B44, Becton Dickinson, San Jose, CA), biotinylated horse anti-mouse (rat absorbed) IgG secondary antibody diluted 1:200 as a linking reagent (BA-2001, Vector Laboratories, Burlingame, CA), peroxidase-conjugated avidin as the label (ABC Complex Vector Kit, Vector Laboratories), and 3,3′-diaminobenzidine as the chromogen (Vector Mouse IgG Standard Kit). A section of duodenum was used as an internal positive control on each slide. Negative and positive control slides were run with each immunostaining batch. BrdU LI was determined by counting approximately 400 TB epithelial cells per slide (mean ± SE, 462 ± 20) across up to seven fields/section by an observer blind to experimental groups. Labeling index was calculated as the percentage of positively labeled cells out of the total cells counted. Bronchioles with clear evidence of inflammation were not counted.

### Apoptosis and inflammation markers

Total RNA was isolated from homogenized right cranial lung lobes from rats in the 10-day exposure study using silica membrane spin columns (RNeasy mini-kit, Qiagen, Valencia, CA). Following addition of RNAse inhibitor, RNA yield was determined spectrophotometrically (NanoDrop 1000, Thermo Scientific, Wilmington, DE). Real-time quantitative reverse transcription PCR (RT-qPCR) was performed in duplicate samples (25 ng total RNA) using SuperScript III One-step RT-qPCR kits (Invitrogen, Grand Island, NY), probe (TaqMan 5′-labeled 6-carboxyfluorescein amidite), and primers for rat *Nlrp3*, *Pycard*, *Casp1*, *Il1b*, *Il6, Il18*, *Tnfa*, *Ifng*, *Cxcl2*, and 18S rRNA as an internal control for each sample (primers and probe from Applied Biosystems Inc., Foster City, CA). RT-qPCR for each transcript was run separately on an ABI Prism 7900 HT sequence detection system (Applied Biosystems Inc., Foster City, CA) under the following conditions: 20 min at 53 °C (reverse transcription), 2 min at 95 °C (inactivation of reverse transcriptase), followed by 40 cycles of 45 s at 60 °C and 15 s at 95 °C. Data were analyzed using ABI sequence detection software (version 2.2). For each PCR plate, cycle threshold (Ct) was set to an order of magnitude above background, and each sample target gene Ct was normalized to control 18S Ct. Expression in each exposure group was quantified as percent of the air control group.

### Akt pathway, MAPK/ERK cascade, and inflammatory cytokines

Right cranial lung lobes from rats necropsied 1 day, 1, and 3 months after the 13-week exposure were homogenized in Tris lysis buffer containing phosphatase and protease inhibitors and phenylmethylsulfonyl fluoride (Meso Scale Discovery (MSD), Gaithersburg, MD), and analyzed for total protein. Multi-Spot® 96-well plates (MSD) pre-coated with rat-specific capture antibodies to proteins in the Akt pathway or the MAPK/ERK cascade (both in 4-spot formats) were prepared with blocking solution (MSD), and samples were added at equal protein levels to each well. Activated (phosphorylated) components measured in the Akt signaling pathway included pAkt, p70S6K, pS6RP, and pGSK-3β. Components measured in the MAPK/ERK cascade included pERK1/2, pMEK1/2, and pSTAT3. Inflammatory and allergic cytokines in lung samples and calibrated cytokine standards were measured in a blocked Multi-Spot® 96-well 7-spot plate (MSD) pre-coated with rat-specific capture antibodies to IL-1β, IL-4, IL-5, IL-13, IFN-γ, TNF-α, and CXCL1. Plate washing, addition of detection antibodies, and electrochemilumiscence detection on a Sector Imager 2400 (MSD) were performed according to manufacturer’s instructions. Activities in Akt and MAPK/ERK pathways were quantified in each exposure group as percent of the air control group. Inflammatory and allergic cytokine levels were expressed relative to total protein in the lung homogenate (pg/mg protein).

### Statistical analysis

Statistical analyses were performed using Prism 6 (GraphPad Software), STATISTIX for Windows v2.0 (Analytical Software, Tallahassee, FL), JMP v8.0 (Cary, NC), and SAS statistical package v9.4 (SAS Institute, Cary, NC). Variance of continuous data was tested using Levene’s test, Welch’s ANOVA, or a standard ANOVA. Subsequent pairwise comparisons were made using a Tukey-Kramer’s Honestly Significant Difference *post hoc* test unless otherwise indicated. Cell proliferation data (BrdU LI) were screened for distribution and homogeneity of variance and analyzed using ANOVA followed by pairwise tests of least square means between control and fiber-exposed groups. Pairwise *P* values were adjusted using a Bonferroni multiple-comparison correction. A linear test for trend was also applied to BrdU LI values among LA dose groups. For histopathology data, a one-sided Fisher’s exact test was used to evaluate group differences in lesion incidence, and a Bonferroni correction was applied to all pairwise *P* values. A dose response trend for LA exposure was evaluated using a Cochran-Armitage trend test on lung tumor and hyperplasia incidence at 18 months post-exposure. For tumors, pairwise and trend tests were run using all animals with pathology data (*n* = 49–50/group) and using a time-adjusted reduced analysis in which early deaths that occurred prior to the time at which the first lung tumor was observed were excluded (*n* = 41–44/group) [69]. Group differences or trend tests with an adjusted *P* value < 0.05 were considered statistically significant.

#### Availability of data and material

Data supporting the findings is found in the main paper and additional supporting file. Raw data files will also be shared by the corresponding author upon request.

## Additional file

Additional file 1:
**AF Table S1.** Complete blood cell analysis in rats exposed to AM or LA for 13 weeks and necropsied 1 day, 1 month, 3 months, or 18 months after the end of the exposure. **AF Table S2.** Likely cause of early death or moribund sacrifice in the 18-month post- exposure arm. **AF Table S3.** Histopathology findings in the left epididymides and testes of rats 18 months following exposure to AM or LA for 13 weeks. **AF Figure S1.** EDS analysis of representative fibers in lung tissues from rats exposed to AM (top) or LA (bottom) and necropsied 18 months after exposure. **AF Figure S2.** Long-term stop-exposure inhalation study: survival of rats by treatment groups following exposure to AM or LA for 13 weeks. **AF Figure S3.** Diagram of fiber exposure system. (DOCX 268 kb)

## Abbreviations

ALP: alkaline phosphatase
AM: amosite
ANOVA: analysis of variance
APS: aerosol particle sizer
ASC: apoptosis-associated speck-like protein containing a CARD
B/A: bronchiolar/alveolar
BALF: bronchoalveolar lavage fluid
BrdU: 5-bromo-2′-deoxyuridine
CARD: caspase recruitment domain
Casp1: caspase-1
Ct: cycle threshold
*Ctsb*: cathepsin B
CXCL1: chemokine (C-X-C motif) ligand 1
*Cxcl2*: chemokine (C-X-C motif) ligand 2
CXCR2: C-X-C chemokine receptor 2
D: diameter
EDS: energy-dispersive x-ray spectroscopy
EPA: U.S. Environmental Protection Agency
EPL: Experimental Pathology Laboratories
EDTA: ethylenediaminetetraacetate
H&E: hematoxylin and eosin
HEPA: high-efficiency particulate air
Hct: hematocrit
Hgb: hemoglobin
*Ifng* (IFN-γ): interferon-gamma
Il: interleukin
IN: intranasal
IT: intratracheal
KC/GRO: keratinocyte chemoattractant/ growth-related oncogene
L: length
LA: Libby amphibole
LDH: lactate dehydrogenase
LI: labeling index
LRT: lower respiratory tract
MAPK/ERK: mitogen-activated protein kinases/extracellular signal-regulated kinases
MC: mean corpuscular
MCH: mean corpuscular hemoglobin
MCHC: mean corpuscular hemoglobin concentration
MCL: mononuclear cell leukemia
MCV: mean corpuscular volume
MOA: mode of action
MSD: meso scale discovery
NAG: N-acetyl β-D glucosaminidase
NBF: neutral buffered formalin
NALP3: NACHT, LRR and PYD domains-containing protein 3
*Nlrp3*: NOD-like receptor family, pyrin domain containing 3
NTP: National Toxicology Program
p70S6K: phospho-70 kDa ribosomal protein S6 kinase (Thr389)
pAkt: phospho-Akt (Ser473)
PDK1: phosphoinositide-dependent kinase 1
pERK1/2: phospho-extracellular signal-regulated kinase 1, 2 (Thr202/Thr204; Thr185/Tyr187)
pGSK-3β: phospho-glycogen synthase kinase-3β (Ser9)
pMEK1/2: phospho-mitogen-activated protein ERK kinase 1, 2 (Ser217/221)
pS6RP: phospho-S6 ribosomal protein (Ser240/244)
pSTAT3: phospho-signal transducer and activator of transcription 3 (Tyr705)
Pycard (PYCARD): pyrin domain caspase recruitment domain
RAM: respirable aerosol monitor
RBC: red blood cells
RT-qPCR: quantitative reverse transcription PCR
SEM: scanning electron microscope
TB: terminal bronchiole
*Tnfa* (TNF-α): tumor necrosis factor-alpha
URT: upper respiratory tract
WBC: white blood cells
WHO: World Health Organization
