# Supplementary material for: Persistent effects of Libby amphibole and amosite asbestos following subchronic inhalation in rats
Source: Part Fibre Toxicol. 2016 Apr 15;13:17. doi: 10.1186/s12989-016-0130-z (PMC4832450; doi:10.1186/s12989-016-0130-z)
Supplement: Additional file 1: — AF Table S1. Complete blood cell analysis in rats exposed to AM or LA for 13 weeks and necropsied 1 day, 1 month, 3 months, or 18 months after the end of the exposure. AF Table S2. Likely cause of early death or moribund sacrifice in the 18-month post- exposure arm. AF Table S3. Histopathology findings in the left epididymides and testes of rats 18 months following exposure to AM or LA for 13 weeks. AF Figure S1. EDS analysis of representative fibers in lung tissues from rats exposed to AM (top) or LA (bottom) and necropsied 18 months after exposure. AF Figure S2. Long-term stop-exposure inhalation study: survival of rats by treatment groups following exposure to AM or LA for 13 weeks. AF Figure S3. Diagram of fiber exposure system. (DOCX 268 kb) [file 12989_2016_130_MOESM1_ESM.docx]

**ADDITIONAL FILE 1**

**Persistent Effects of Libby Amphibole and Amosite Asbestos**

**Following Subchronic Inhalation in Rats**

Stephen H. Gavett, Carl U. Parkinson, Gabrielle A. Willson, Charles E. Wood,

Annie M. Jarabek, Kay C. Roberts, Urmila P. Kodavanti, and Darol E. Dodd

**AF Table S1**. Complete blood cell analysis in rats exposed to AM or LA for 13 weeks and evaluated 1 day, 1 month, 3 months, or 18 months after the end of the exposure. Top row represents target mass concentrations (mg/m^3^). Results show means ± SE of 8 rats/group.

|  | **Air Control** | **AM 3.3** | **LA 1.0** | **LA 3.3** | **LA 10.0** |
| --- | --- | --- | --- | --- | --- |
| WBC (x 10^3^/μl)  1 Day  1 Month  3 Months  18 Months | 3.4 ± 0.3  4.3 ± 0.3  3.7 ± 0.3  3.3 ± 0.4 | 3.4 ± 0.1  4.7 ± 0.7  3.3 ± 0.2  3.0 ± 0.3 | 4.0 ± 0.3  3.9 ± 0.2  3.5 ± 0.3  3.1 ± 0.2 | 3.2 ± 0.3  4.2 ± 0.2  3.4 ± 0.4  3.6 ± 0.3 | 3.7 ± 0.2  4.2 ± 0.3  4.0 ± 0.5  4.4 ± 0.7 |
| Lymphocytes (x 10^3^/μl)  1 Day  1 Month  3 Months  18 Months | 2.5 ± 0.2  3.6 ± 0.3  2.4 ± 0.2  1.6 ± 0.1 | 2.2 ± 0.1  3.7 ± 0.4  2.1 ± 0.1  1.4 ± 0.1 | 2.5 ± 0.2  3.3 ± 0.2  2.3 ± 0.1  1.5 ± 0.1 | 2.2 ± 0.2  3.3 ± 0.1  2.3 ± 0.2  1.7 ± 0.2 | 2.4 ± 0.1  3.3 ± 0.3  2.3 ± 0.3  1.9 ± 0.2 |
| Lymphocytes (% WBC)  1 Day  1 Month  3 Months  18 Months | 73.3 ± 1.6  83.7 ± 1.9  63.8 ± 2.5  51.4 ± 3.5 | 64.2 ± 1.2*  81.5 ± 2.8  62.7 ± 2.8  47.3 ± 3.2 | 61.5 ± 2.1*§  81.9 ± 1.2  67.4 ± 1.9  49.0 ± 2.9 | 68.0 ± 1.2  79.5 ± 2.7  67.5 ± 1.5  46.1 ± 2.4 | 64.6 ± 0.9*  78.4 ± 3.4  58.1 ± 1.4#§  40.8 ± 3.8 |
| RBC (x 10^6^/μl)  1 Day  1 Month  3 Months  18 Months | 8.1 ± 0.3  8.4 ± 0.1  7.9 ± 0.2  7.6 ± 0.2 | 8.1 ± 0.1  8.4 ± 0.1  7.7 ± 0.1  7.6 ± 0.1 | 8.2 ± 0.1  8.3 ± 0.1  7.6 ± 0.1  7.8 ± 0.3 | 8.3 ± 0.1  8.4 ± 0.1  7.6 ± 0.1  7.8 ± 0.1 | 8.2 ± 0.1  8.4 ± 0.1  7.7 ± 0.1  7.1 ± 0.2 |
| Platelets (x 10^3^/μl)  1 Day  1 Month  3 Months  18 Months | 517 ± 40  634 ± 24  479 ± 34  604 ± 48 | 520 ± 16  539 ± 27  471 ± 27  538 ± 54 | 580 ± 25  661 ± 36  548 ± 22  603 ± 30 | 537 ± 18  559 ± 17  564 ± 53  612 ± 35 | 496 ± 34  489 ± 38*#  557 ± 26  685 ± 50 |
| Hgb (g/dL)  1 Day  1 Month  3 Months  18 Months | 14.4 ± 0.5  14.4 ± 0.2  14.9 ± 0.3  14.8 ± 0.5 | 14.6 ± 0.1  14.3 ± 0.2  14.3 ± 0.1  14.8 ± 0.2 | 14.8 ± 0.2  14.3 ± 0.2  14.3 ± 0.1  15.2 ± 0.5 | 14.8 ± 0.1  14.4 ± 0.2  14.2 ± 0.1*  15.2 ± 0.2 | 14.5 ± 0.2  14.2 ± 0.2  14.4 ± 0.2  13.8 ± 0.5 |
| Hct (%)  1 Day  1 Month  3 Months  18 Months | 40.8 ± 1.7  43.6 ± 0.8  39.7 ± 0.9  41.0 ± 1.5 | 41.0 ± 0.5  43.3 ± 0.6  37.8 ± 0.5  41.5 ± 0.7 | 41.6 ± 0.5  43.3 ± 0.8  38.1 ± 0.4  42.5 ± 1.4 | 42.1 ± 0.4  43.3 ± 0.8  37.7 ± 0.4  42.0 ± 0.7 | 41.4 ± 0.6  43.0 ± 0.7  38.2 ± 0.5  38.4 ± 1.5 |
| MCH (pg/cell)  1 Day  1 Month  3 Months  18 Months | 17.9 ± 0.2  17.3 ± 0.2  18.9 ± 0.1  19.4 ± 0.2 | 17.9 ± 0.2  17.1 ± 0.3  18.5 ± 0.1  19.6 ± 0.1 | 18.0 ± 0.2  17.1 ± 0.2  18.8 ± 0.1  19.5 ± 0.2 | 17.7 ± 0.1  17.1 ± 0.1  18.7 ± 0.1  19.5 ± 0.2 | 17.6 ± 0.1  16.9 ± 0.1  18.7 ± 0.1  19.4 ± 0.2 |
| MCHC (g/dL)  1 Day  1 Month  3 Months  18 Months | 35.4 ± 0.4  33.0 ± 0.4  37.6 ± 0.3  36.1 ± 0.1 | 35.6 ± 0.4  32.9 ± 0.3  37.7 ± 0.3  35.8 ± 0.1 | 35.5 ± 0.3  33.0 ± 0.2  37.6 ± 0.3  35.8 ± 0.2 | 35.2 ± 0.2  33.1 ± 0.2  37.6 ± 0.3  36.1 ± 0.2 | 35.0 ± 0.2  33.1 ± 0.3  37.6 ± 0.3  36.1 ± 0.3 |
| MCV (fL)  1 Day  1 Month  3 Months  18 Months | 50.6 ± 0.3  52.1 ± 0.5  50.3 ± 0.1  53.6 ± 0.7 | 50.3 ± 0.2  51.8 ± 0.4  49.2 ± 0.2*  54.7 ± 0.3 | 50.8 ± 0.2  51.9 ± 0.4  50.0 ± 0.1  54.6 ± 0.4 | 50.5 ± 0.1  51.7 ± 0.4  49.6 ± 0.3  54.1 ± 0.6 | 50.2 ± 0.2  50.9 ± 0.3  49.8 ± 0.2  53.7 ± 0.8 |

**P* < 0.05 vs. air control; # vs. LA mg/m^3^, § vs. LA mg/m^3^.

**AF Table S2**. Likely cause of early death or moribund sacrifice in the 18-month post exposure arm.

|  | **Air Control** | **AM 3.3** | **LA 1.0** | **LA 3.3** | **LA 10.0** |
| --- | --- | --- | --- | --- | --- |
| **No. started on study** | **50** | **50** | **50** | **50** | **50** |
| **No. of early death/sacrifice** | **10** | **10** | **19** | **9** | **12** |
| Mononuclear cell leukemia | 3 | 5 | 5 | 1 | 7 |
| Oral mucosa squamous carcinoma | 2 | 3 | 4 | 1 | 0 |
| Pituitary adenoma | 2 | 1 | 2 | 1 | 0 |
| Peritoneal mesothelioma | 0 | 0 | 2 | 1 | 0 |
| Nephropathy | 0 | 0 | 1 | 0 | 2 |
| Undetermined | 0 | 1 | 1 | 1 | 0 |
| Preputial gland carcinoma | 0 | 0 | 0 | 2 | 0 |
| Osteosarcoma | 1 | 0 | 1 | 0 | 0 |
| Salivary gland carcinoma | 1 | 0 | 0 | 0 | 0 |
| Subcutis fibroma | 1 | 0 | 0 | 0 | 0 |
| Mesentery schwannoma | 0 | 0 | 1 | 0 | 0 |
| Thyroid follicular cell carcinoma | 0 | 0 | 1 | 1 | 0 |
| Lung alveolar/bronchiolar carcinoma | 0 | 0 | 0 | 0 | 1 |
| Mammary gland fibroadenoma | 0 | 0 | 0 | 0 | 1 |
| Meningioma | 0 | 0 | 0 | 0 | 1 |
| Adrenal cortex carcinoma | 0 | 0 | 0 | 1 | 0 |
| Subcutis sarcoma | 0 | 0 | 1 | 0 | 0 |

**AF Table S3**. Histopathology findings in the left epididymides and testes of rats 18 months following exposure to AM or LA for 13 weeks. Values shown are the number of animals with a finding in each group. Average severity score for all rats in each group are shown in parentheses.^1^

|  | **Air Control** | **AM 3.3** | **LA 1.0** | **LA 3.3** | **LA 10.0** |
| --- | --- | --- | --- | --- | --- |
| Epididymis (left) |  |  |  |  |  |
| **No. Examined** | **49** | **50** | **50** | **49** | **50** |
| Hypospermia | 28 (1.9) | 36 (2.6) | 27 (1.7) | 25 (1.7) | 26 (1.9) |
| Sperm granuloma | 1 | 0 | 0 | 0 | 0 |
| Arteritis | 0 | 0 | 1 | 0 | 0 |
| Testis (left) |  |  |  |  |  |
| **No. Examined** | **49** | **50** | **49** | **49** | **50** |
| Tubular atrophy | 25 (0.8) | 16 (0.8) | 15 (0.6) | 12 (0.4) | 14 (0.5) |
| Edema | 13 (0.5) | 6 (0.2) | 4 (0.2) | 8 (0.2) | 8 (0.2) |
| Interstitial cell adenoma | 31 | 38 | 34 | 33 | 37 |
| Interstitial cell hyperplasia | 0 (0.0) | 5 (0.2) | 1 (0.1) | 1 (0.0) | 1 (0.1) |
| Mesothelioma | 2 | 0 | 3 | 2 | 0 |

^1^Lesion severity score was graded as follows: 1=minimal, 2=mild, 3=moderate, 4=marked, and 5=severe.

**AF Figure S1**. EDS analysis of representative fibers in lung tissues from rats exposed to AM (top) or LA (bottom) and necropsied 18 months after exposure. Analysis confirmed the chemistry of the fibers was consistent with either AM or LA.

AM – 18 month lung

LA – 18 month lung

**AF Figure S2**. Long-term stop-exposure inhalation study: survival of rats by treatment groups following exposure to AM or LA for 13 weeks. Numbers in legend represent target mass concentrations (mg/m^3^). Average survival time was not significantly different among groups.


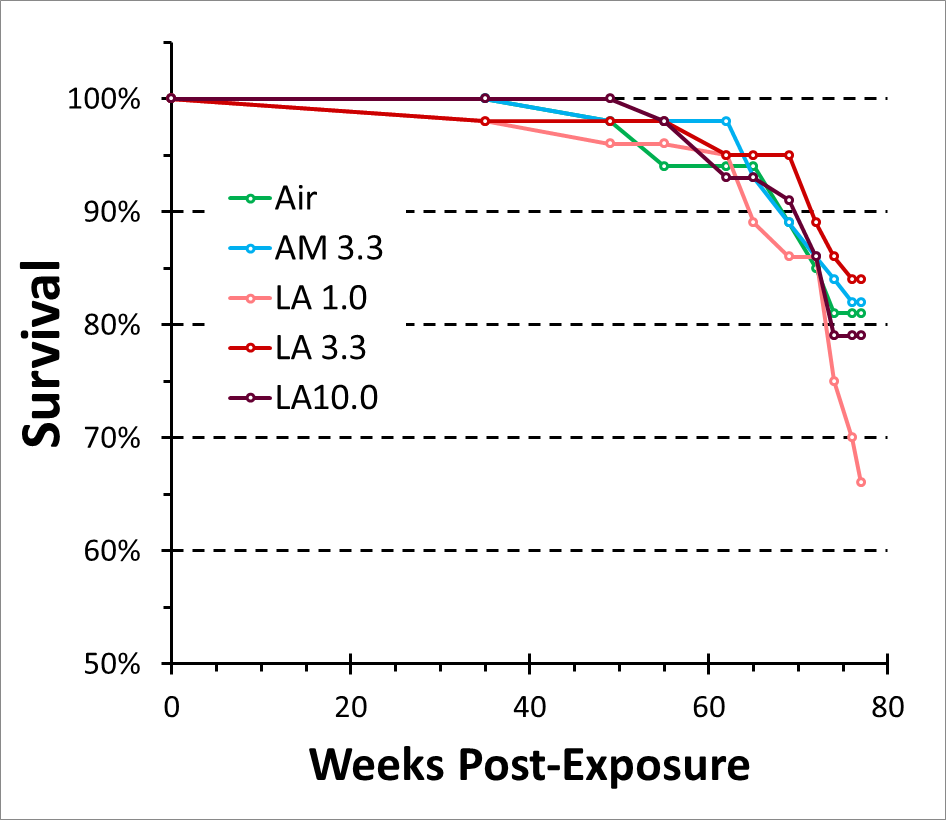


**AF Figure S3**. Diagram of fiber exposure system. See Methods-Exposure system for details. The control (air-only) exposure system was similar in design, but consisted of only six tiers.
